# Impact of the Oral Adsorbent AST-120 on Organ-Specific Accumulation of Uremic Toxins: LC-MS/MS and MS Imaging Techniques

**DOI:** 10.3390/toxins10010019

**Published:** 2017-12-28

**Authors:** Emiko Sato, Daisuke Saigusa, Eikan Mishima, Taeko Uchida, Daisuke Miura, Tomomi Morikawa-Ichinose, Kiyomi Kisu, Akiyo Sekimoto, Ritsumi Saito, Yuji Oe, Yotaro Matsumoto, Yoshihisa Tomioka, Takefumi Mori, Nobuyuki Takahashi, Hiroshi Sato, Takaaki Abe, Toshimitsu Niwa, Sadayoshi Ito

**Affiliations:** 1 Division of Clinical Pharmacology and Therapeutics, Tohoku University Graduate School of Pharmaceutical Sciences, Sendai 980-8578, Japan; altosax_sakura_thanklsuccess@yahoo.co.jp (T.U.); take-seki@med.tohoku.ac.jp (A.S.); ntakaha@m.tohoku.ac.jp (N.T.); hsymhs2i@m.tohoku.ac.jp (H.S.); 2 Division of Nephrology, Endocrinology and Vascular Medicine, Tohoku University Graduate School of Medicine, Sendai 980-8574, Japan; eikan@med.tohoku.ac.jp (E.M.); ki30126@med.tohoku.ac.jp (K.K.); tmori@hosp.tohoku-mpu.ac.jp (T.M.); db554@med.tohoku.ac.jp (S.I.); 3 Department of Integrative Genomics, Tohoku Medical Megabank Organization, Tohoku University, Sendai 980-8573, Japan; d.saigusa@gmail.com (D.S.); ritsumi.saito.e5@tohoku.ac.jp (R.S.); 4 Innovation Center for Medical Redox Navigation, Kyushu University, Fukuoka 812-8582, Japan; daipon@agr.kyushu-u.ac.jp (D.M.); ichinose226@gmail.com (T.M.-I.); 5 Division of Feto-Maternal Medical Science, Department of Community Medical Support, Tohoku Medical Megabank Organization, Tohoku University, Sendai 980-8574, Japan; yuji-oe@med.tohoku.ac.jp; 6 Division of Oncology, Pharmacy Practice and Sciences, Tohoku University Graduate School of Pharmaceutical Sciences, Sendai 980-8578, Japan; matsumotoy@m.tohoku.ac.jp (Y.M.); ytomioka@m.tohoku.ac.jp (Y.T.); 7 Division of Integrative Renal Replacement Therapy, Tohoku University Graduate School of Medicine, Sendai 980-8574, Japan; 8 Division of Medical Science, Tohoku University Graduate School of Biomedical Engineering, Sendai 980-8574, Japan; takaabe@med.tohoku.ac.jp; 9 Shubun University, Ichinomiya 491-0938, Japan; niwa.t@shubun.ac.jp

**Keywords:** uremic toxin, chronic kidney disease, indoxyl sulfate, *p*-cresyl sulfate, mass spectrometry

## Abstract

Elevated circulating uremic toxins are associated with a variety of symptoms and organ dysfunction observed in patients with chronic kidney disease (CKD). Indoxyl sulfate (IS) and *p*-cresyl sulfate (PCS) are representative uremic toxins that exert various harmful effects. We recently showed that IS induces metabolic alteration in skeletal muscle and causes sarcopenia in mice. However, whether organ-specific accumulation of IS and PCS is associated with tissue dysfunction is still unclear. We investigated the accumulation of IS and PCS using liquid chromatography/tandem mass spectrometry in various tissues from mice with adenine-induced CKD. IS and PCS accumulated in all 15 organs analyzed, including kidney, skeletal muscle, and brain. We also visualized the tissue accumulation of IS and PCS with immunohistochemistry and mass spectrometry imaging techniques. The oral adsorbent AST-120 prevented some tissue accumulation of IS and PCS. In skeletal muscle, reduced accumulation following AST-120 treatment resulted in the amelioration of renal failure-associated muscle atrophy. We conclude that uremic toxins can accumulate in various organs and that AST-120 may be useful in treating or preventing organ dysfunction in CKD, possibly by reducing tissue accumulation of uremic toxins.

## 1. Introduction

Uremic toxins accumulate in the circulation with the progression of chronic kidney disease (CKD). Because the accumulated uremic toxins exert deleterious effects, they increase morbidity in CKD. Beyond affecting the kidney, retention of uremic toxins is involved in the various complications that occur in CKD, including hypertension, cardiovascular diseases, neurological impairment, bone disorders, and muscle wasting syndrome [1,2,3,4,5].

Indoxyl sulfate (IS) and *p*-cresyl sulfate (PCS) are well-studied representative uremic toxins with cytotoxic, pro-inflammatory, and pro-fibrotic effects [5,6,7,8,9]. IS and PCS induce oxidative stress and pro-inflammatory effects in the kidney. The pro-inflammatory effect of IS occurs at least partly through activation of the aryl hydrocarbon receptor (AhR)/NF-κB pathway. IS is an endogenous agonist for AhR [10], and AhR signaling can activate inflammatory responses [6].

In addition, IS and PCS exert adverse effects on a variety of cells, such as cardiac myocytes, osteoblasts, skeletal myocytes, and neuronal cells, contributing to cardiac fibrosis, mineral and bone disorders, insulin resistance, and cognitive impairment in CKD [11,12,13,14,15,16]. We recently reported the effect of IS on skeletal muscle. IS causes uremic muscle atrophy in CKD by inducing metabolic alterations as an antioxidative stress response [17]. Thus, drugs that can reduce the levels of circulating IS and PCS, such as AST-120, an oral adsorbent, are considered a therapeutic option for prevention of uremia and its related symptoms in CKD.

IS enters various cells via the organic anion transporters (OATs) OAT1 and OAT3 [18,19,20,21], and our previous report demonstrated that IS alters mitochondrial metabolism [17]. Accordingly, organ dysfunction resulting from accumulation of uremic toxins inside cells may be a more harmful consequence than uremic toxin accumulation in the circulation of CKD patients. However, the levels of accumulated uremic toxins in various organs have not been quantified.

The aim of the present study was to determine the levels of IS and PCS in various organs using a mouse model of renal failure (RF) and liquid chromatography-tandem mass spectrometry (LC-MS/MS), and to visualize the spatial distribution of uremic toxins in tissue specimens using mass spectrometry imaging (MSI). Additionally, we evaluated the mitigating effect of AST-120 on tissue accumulation of uremic toxins in various organs.

## 2. Results

### 2.1. Characteristics of the RF Mouse Model

**Figure 1:**
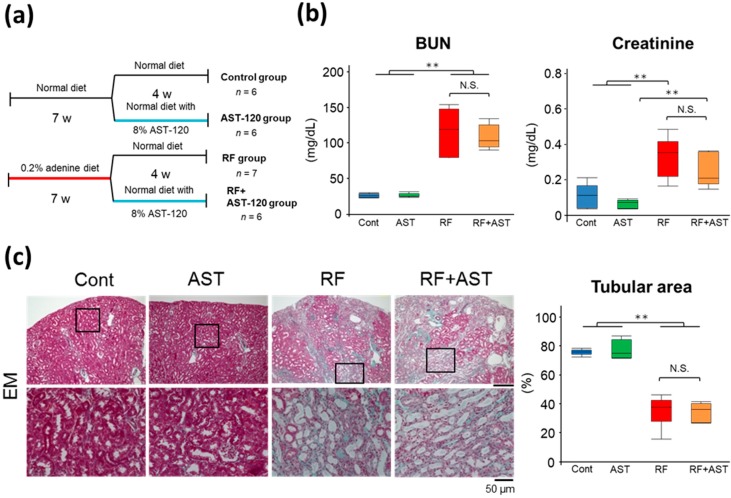
Renal histology and function in adenine-induced renal failure mice. (**a**) Experimental schedule. (**b**) Blood urea nitrogen (BUN) and plasma creatinine concentrations. *n* = 5–7 per group. (**c**) Representative histologic images of Elastica-Masson (EM) staining of kidney sections. Morphometric analysis of the percentage of the remaining cortical tubular area. The cortical tubular area was calculated from the EM staining images. Data are expressed as box plots. Tukey-Kramer test: ** *p* < 0.01. Cont, control; AST, AST-120; RF, renal failure; RF + AST, RF mice treated with AST-120; N.S., not significant.

Because AST-120 is used clinically in patients with CKD, we first tested the effect of AST-120 on renal function and morphological changes in kidneys from adenine-induced CKD mice. We used the following four groups of mice: (1) Control (*n* = 6), (2) AST-120 (*n* = 6), (3) RF (*n* = 7), and (4) RF + AST-120 (*n* = 6) (Figure 1a). RF was induced by a 7-week oral treatment with adenine. The total body weight and the weights of the kidneys, brain, white adipose tissue (WAT), and testis were significantly lower in the RF and RF + AST-120 groups than in Control and AST-120 mice (Table 1).

Next, we examined renal function and histological changes in the mice. The levels of blood urea nitrogen (BUN) and creatinine were 4.5- and 3.2-fold higher, respectively, in the RF group than in the control group; there were no significant differences in these parameters between the RF and RF + AST-120 group (Figure 1b). Although severe renal fibrosis and tubular damage were observed in both the RF and RF + AST-120 groups (Figure 1c), their levels were not significantly affected by AST-120 treatment (Figure 1c). These results suggest that AST-120 does not affect renal function and morphological changes in RF, at least under our experimental conditions, in which severe renal damage had been induced by adenine before the start of AST-120 treatment.

### 2.2. IS and PCS Accumulated in All Organs Tested

**Figure 2:**
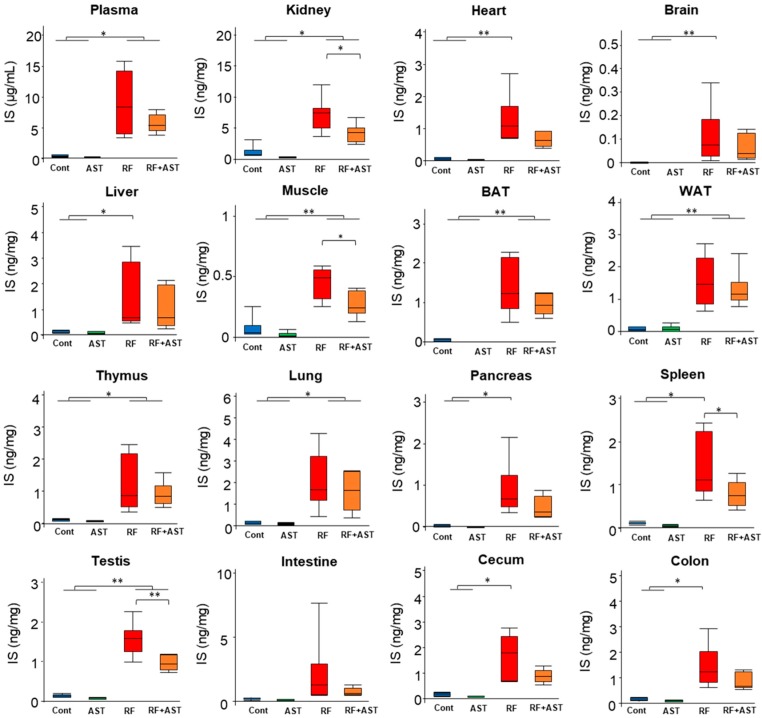
Indoxyl sulfate (IS) accumulation in plasma and tissues. Comparison of plasma and tissue IS levels. Data are expressed as box plots. *n* = 6–7 per group. Tukey-Kramer test: * *p* < 0.05, ** *p* < 0.01. Cont, control; AST, AST-120; RF, renal failure; RF + AST, RF mice treated with AST-120; BAT, brown adipose tissue; WAT, white adipose tissue.

**Figure 3:**
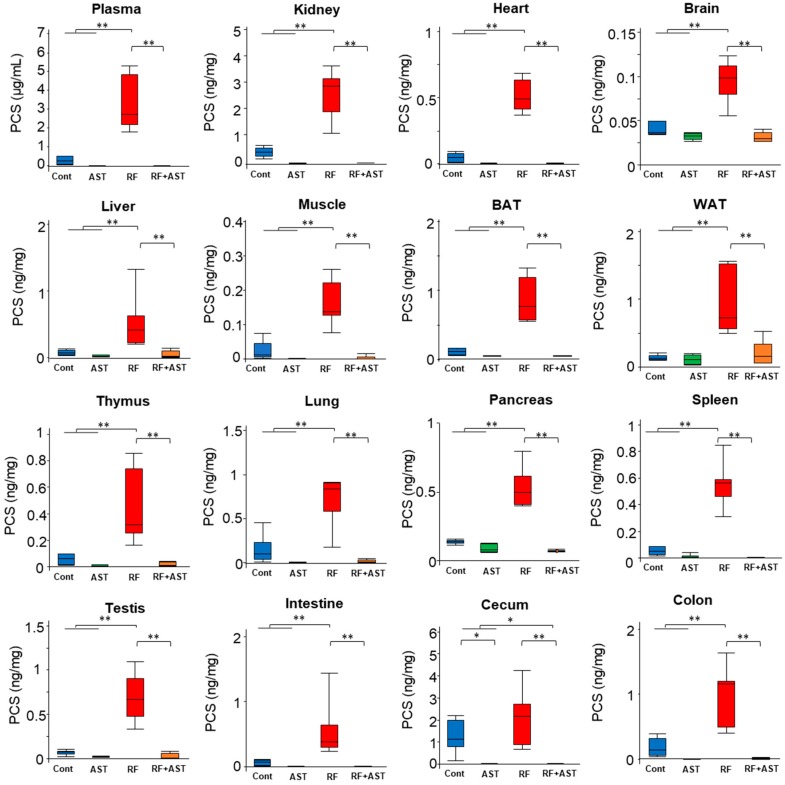
*p*-Cresyl sulfate (PCS) accumulation in plasma and tissues. Comparison of plasma and tissue PCS levels. Data are expressed as box plots. *n* = 6–7 for each group. Tukey-Kramer test: * *p* < 0.05, ** *p* < 0.01. Cont, control; AST, AST-120; RF, renal failure; RF + AST, RF mice treated with AST-120; BAT, brown adipose tissue; WAT, white adipose tissue.

Although we were not able to detect a significant beneficial effect of AST-120 in this study, this does not exclude potential effects of AST-120 on other parameters in the kidney or other tissues, or on tissue accumulation of uremic toxins. Accordingly, we tested tissue accumulation of the uremic toxins IS and PCS in the four experimental groups. Levels of IS and PCS in plasma and in 15 systemic organs were measured by LC-MS/MS (Figure 2 and Figure 3). Tissues were perfused before harvesting to flush out blood. IS plasma levels were 20-fold higher in the RF group than in the control group. AST-120 treatment in the RF mice did not significantly reduce elevated plasma IS levels (Figure 2). Similarly, IS levels in the RF group were significantly higher than in the control group for all 15 organs analyzed. IS levels in the kidney, muscle, brain, heart, brown adipose tissue (BAT), WAT, liver, thymus, lung, pancreas, spleen, testis, small intestine, cecum, and colon were 10.8-, 6.2-, 184-, 18.9-, 48.3-, 23.0-, 12.0-, 11.1-, 11.7-, 43.1-, 13.3-, 11.6-, 11.5-, 9.0-, and 11.0-fold higher in the RF group than in the control group. Among these organs, the highest IS levels were observed in the kidney. AST-120 treatment in the RF mice significantly reduced elevated IS levels in the kidney (41.6% compared with the RF group, *p* < 0.01), muscle (35.2%, *p* < 0.05), spleen (46.4%, *p* < 0.05), and testis (36.8%, *p* < 0.01).

PCS accumulation in plasma and organs was also measured by LC-MS/MS (Figure 3). Plasma PCS levels were 12-fold higher in the RF group than in the control group. AST-120 treatment markedly decreased elevated plasma PCS levels in the RF groups (0.4% compared with the RF group, Figure 3). Similarly, significantly higher PCS levels were measured in all organs in the RF group than in the control group. PCS levels in the kidney, muscle, brain, heart, BAT, WAT, liver, thymus, lung, pancreas, spleen, testis, small intestine, cecum, and colon were 5.9-, 7.8-, 2.4-, 10.3-, 7.8-, 7.0-, 7.1-, 7.3-, 7.4-, 7.7-, 10.5-, 10.4-, 8.9-, 1.7-, and 5.6-fold higher in the RF group than in the control group. AST-120 treatment in the RF group decreased elevated PCS levels in all organs analyzed: Kidney (0.3% compared with the RF group, *p* < 0.01), muscle (1.9%, *p* < 0.01), brain (32.9%, *p* < 0.01), heart (1.0%, *p* < 0.01), BAT (6.5%, *p* < 0.01), WAT (21.4%, *p* < 0.01), liver (8.8%, *p* < 0.01), thymus (4.1%, *p* < 0.01), lung (2.9%, *p* < 0.01), pancreas (13.4%, *p* < 0.01), spleen (0.6%, *p* < 0.01), testis (3.6%, *p* < 0.01), small intestine (0.3%, *p* < 0.01), cecum (0.1%, *p* < 0.01), and colon (0.8%, *p* < 0.01). These results demonstrate that IS and PCS accumulated in all systemic organs analyzed, including the brain, and not only in plasma. Moreover, AST-120 treatment reduced the accumulation of IS and PCS in both plasma and organs.

### 2.3. MSI of Uremic Toxins in the Kidney

**Figure 4:**
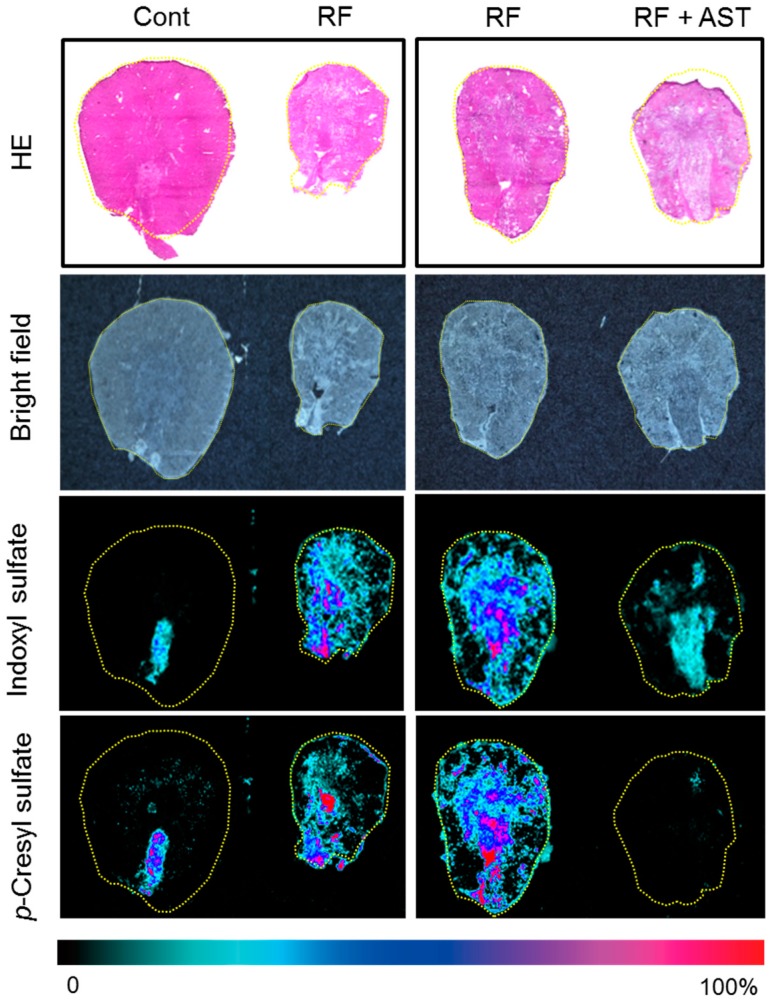
Spatial distribution of indoxyl sulfate (IS) and *p*-cresyl sulfate (PCS) in kidney sections by mass spectrometry imaging. Hematoxylin and eosin (HE) and bright-field images of kidney sections. The second and third RF samples are same sample, but the slide section is different. Mass spectrometry imaging distribution of IS ([M − H]^−^, *m*/*z* 212) and *p*-cresyl sulfate ([M − H]^−^, *m*/*z* 187) in kidney. Cont, control; RF, renal failure; RF + AST, RF mice treated with AST-120.

The levels of IS and PCS in the 15 organs tested do not indicate the spatial distribution of these uremic toxins. The kidneys are composed of various cell types with different functions, and the levels of IS and PCS were higher in the kidneys than in the other organs tested. Accordingly, we next evaluated renal accumulation of IS and PCS by MSI to visualize their spatial distribution. IS and PCS were detected by monitoring ions at *m*/*z* 212, and *m*/*z* 187, respectively. IS was detected only in the renal papilla area in the control group (Figure 4). In contrast, IS signals were detected throughout the kidney in the RF group. The strong signal that indicates accumulation of renal IS was substantially decreased by AST-120 treatment (Figure 4). Similarly to IS, PCS was retained in all parts of the kidney in the RF mice, and its retention was notably decreased by AST-120 treatment (Figure 4). These findings are comparable to those of the LC-MS/MS analysis (Figure 3).

### 2.4. IS Immunohistochemistry and Expression of an Inflammatory Gene in the Kidney

**Figure 5:**
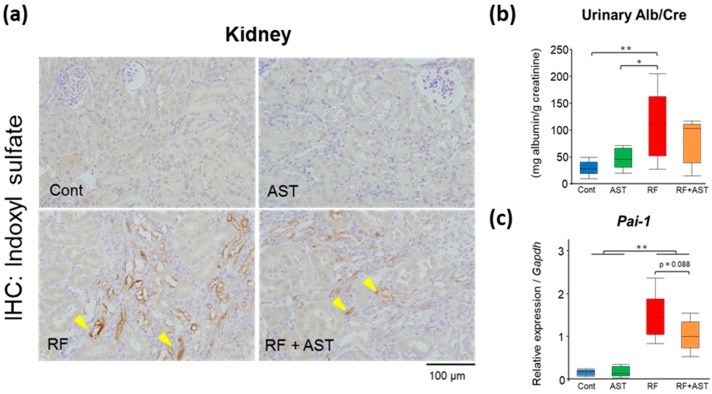
Indoxyl sulfate (IS) immunostaining and expression of an inflammatory gene in kidney. (**a**) Renal immunostaining of IS using an anti-IS antibody. Arrowheads point to immunostaining-positive signals. (**b**) Urinary albumin excretion (creatinine ratio, Alb/Cre) in the four mouse groups. *n* = 5–7 per group. (**c**) Relative mRNA expression levels of plasminogen activator inhibitor 1 (*Pai-1*). Expression levels were normalized to those of *Gapdh*. *n* = 5–7 per group. Data are shown as box plots. Tukey-Kramer test: * *p* < 0.05, ** *p* < 0.01. For *Pai-1* expression, Welch’s *t*-test was performed between the RF and RF + AST groups. Cont, control; AST, AST-120; RF, renal failure; RF + AST, RF mice treated with AST-120; IHC, immunohistochemistry.

To confirm the accumulation and distribution of IS in the kidney of RF mice, we immunostained kidney sections with an anti-IS antibody. We found strong signals in the tubules at the fibrotic area of the RF mice; AST-120 attenuated the IS signal, as shown in the kidneys of the RF + AST-120 mice (Figure 5a), confirming the renal accumulation of IS and its amelioration by AST-120.

Next, we evaluated the effects of AST-120 on albuminuria and expression of a gene involved in renal inflammation. Urinary albumin excretion was 3.9-fold higher in the RF group than in the control group, indicating the glomerular damage in the RF mice. AST-120 treatment resulted in a non-significant reduction in albuminuria in the RF mice (Figure 5b). The expression of a pro-inflammatory gene, plasminogen activator inhibitor 1 (*Pai-1*), was upregulated in the kidneys of the RF mice (Figure 5c), but AST-120 treatment tended to reduce the expression levels. These findings suggest that AST-120 suppresses the accumulation of uremic toxins in renal tissue, contributing to the amelioration of some pathological conditions in the RF kidney.

### 2.5. AST-120 Slows down Muscle Atrophy in RF Mice

**Figure 6:**
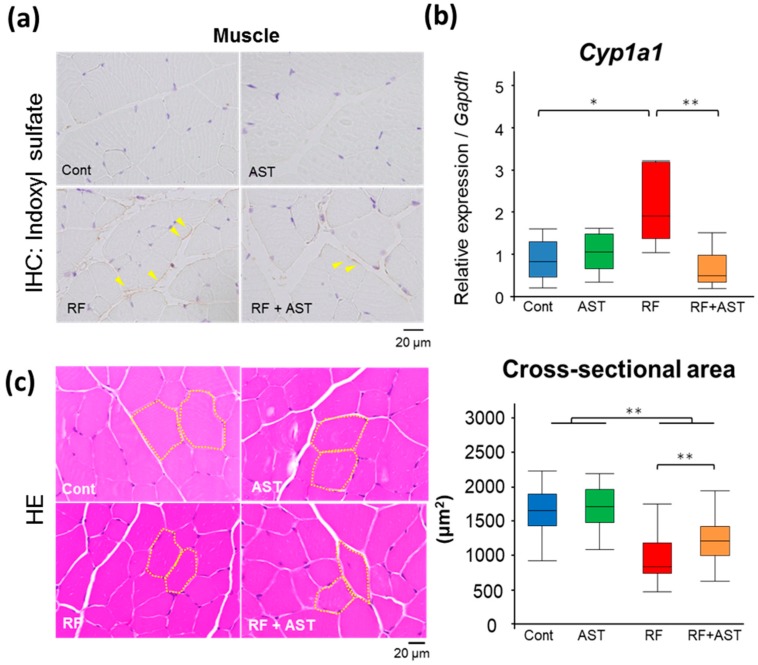
Renal failure-induced skeletal muscle atrophy was ameliorated by AST-120 treatment. (**a**) Immunostaining of indoxyl sulfate (IS) using an anti-IS antibody on the gastrocnemius muscle. Arrowheads point to immunostaining-positive signals. (**b**) Relative mRNA expression levels of cytochrome P450 family 1 subfamily A member 1 (*Cyp1a1*) in skeletal muscle. Expression levels were normalized to those of *Gapdh*. *n* = 5–6 per group. Data are shown as box plots. (**c**) Representative images of hematoxylin and eosin (HE)-stained skeletal muscle sections and cross-sectional area size of the gastrocnemius muscle. Thirty cross-sections were randomly selected from each group for evaluation of cross-sectional area. Data are shown as box plots. Tukey-Kramer test: * *p* < 0.05, ** *p* < 0.01. Cont, control; AST, AST-120; RF, renal failure; RF + AST, RF mice treated with AST-120; IHC, immunohistochemistry.

We recently demonstrated that IS induces sarcopenia in an adenine-induced CKD mouse model [17]. In the current study, immunostaining against IS revealed accumulation of IS in the skeletal muscle from RF mice but not from the control mice (Figure 6a). AST-120 decreased IS accumulation (Figure 6a), consistent with the results of the LC-MS/MS analysis (Figure 3).

Whether the small alterations in the levels of immunopositive IS induced by RF and AST-120 are relevant to the progression of sarcopenia is unclear. IS binds to AhR, activates cytochrome P450 family 1 subfamily A member 1 (*Cyp1a1*), and induces vascular inflammation [22]. We therefore quantified the expression of *Cyp1a1* in skeletal muscle. The skeletal muscle of the RF mice, which contained elevated levels of IS, displayed significantly (*p* < 0.05) higher *Cyp1a1* expression than that of control mice (Figure 6b), suggesting that accumulated IS induced *Cyp1a1* expression. AST-120 restored *Cyp1a1* expression levels in the RF mice to control levels (*p* < 0.01).

We evaluated atrophy in skeletal muscle by analyzing the morphological structure of the gastrocnemius muscle (Figure 6c). The cross-sectional area of skeletal muscle was significantly smaller in the RF mice than in the control group (*p* < 0.01), demonstrating muscle atrophy. Atrophied skeletal muscle in the RF mice was significantly improved by AST-120 treatment (*p* < 0.01). Collectively, these results suggest that the accumulation of uremic toxins in the skeletal muscle resulted in muscle atrophy and harmful effects in RF mice. AST-120 treatment inhibited the retention of uremic toxins, improving the condition of atrophied muscle.

## 3. Discussion

In the present study, we demonstrated that the uremic toxins IS and PCS accumulate in systemic organs and the circulation. High levels of IS accumulated in the BAT, WAT, pancreas, and brain; high levels of PCS accumulated in the testis, spleen, and heart. We also found that AST-120 treatment attenuated IS and PCS accumulation in multiple organs, and contributed to amelioration of pathological conditions in skeletal muscle.

We recently reported that accumulated IS in skeletal muscle induces metabolic alterations by oxidative stress, leading to uremic sarcopenia in CKD [17]. The present findings suggest that tissue accumulation of uremic toxins results in increased *Pai1* expression and AhR signaling, implying the induction of inflammatory processes. As uremic toxins accumulate in whole-body organs, it is plausible that accumulated IS and PCS can induce metabolic alterations, oxidative stress, and inflammation not only in the kidney and skeletal muscle, and may be responsible for CKD-related organ disorders such as a decline in cognitive function, abnormal glucose metabolism, and development of cardiovascular disease.

AST-120 is an orally administered intestinal sorbent consisting of porous carbon particles that are 0.2–0.4 mm in diameter, and was approved for clinical use in Japanese RF patients in 1991 for prolonging the time to initiation of hemodialysis and for improvement of uremic symptoms [23,24]. It adsorbs low-molecular-weight compounds such as indole and *p*-cresol, precursors of IS and PCS, respectively, in the intestines. We found that AST-120 treatment attenuated IS and PCS tissue accumulation not only in the circulation but also in systemic organs. Thus, AST-120 treatment may be expected to ameliorate uremic toxin-related organ disorders in CKD. In the present study, we showed that CKD-related muscle atrophy was inhibited by AST-120. AST-120 has also been shown to improve exercise capacity and mitochondrial biogenesis of skeletal muscle in CKD mice [25]. These results suggest that circulating uremic toxins are transported into whole-body organs and induce aberrations in CKD, and their reduction contributes to amelioration of these aberrations. IS and PCS have both been shown to affect cardiomyocytes. IS was reported to contribute to the development of left ventricular hypertrophy, which is a common complication in CKD [26]. IS levels are associated with cardiovascular events [27]. PCS also contributes to cardiac dysfunction by facilitating cardiac apoptosis [28]. Therefore, reduction of uremic toxins in various organs by AST-120 treatment may ameliorate some CKD-related complications.

MSI is a promising tool for biomedical applications such as biomarker discovery, tissue classification, and drug monitoring, because it provides a label-free and non-staining approach for high-resolution imaging [29]. In the present study, we assessed the changes in renal IS levels using both MSI and immunohistochemistry. MSI also enabled us to evaluate the accumulation and distribution of PCS, for which an antibody has not been developed. Liu et al. reported recently that MSI is effective in analyzing metabolites in RF [30]. We showed that MSI is effective in assessing the distribution of uremic toxins. However, detection of uremic toxins in organs other than kidney and muscle is limited at this time, because of MSI constraints in sensitivity, sample preparation, and image resolution. The technology should be developed further to overcome these technical limitations.

Uptake of uremic toxins into tissues is believed to involve transport through OATs. IS and PCS, which are hydrophobic organic anions, are physiological substrates for OAT families. In the kidney, OATs localized in the basolateral membrane of the proximal tubules are responsible for intracellular uptake of IS and PCS from blood [31]. The transported toxins accumulate and cause the production of free radicals, resulting in nephrotoxicity [18,20,31,32]. OATs are also reportedly expressed in the blood-brain barrier [21], in muscle [19], and in bone osteoblasts [33]. In the present study, we confirmed the expression of OATs (*Slc22a6* and *Slc22a8*) mRNA in the kidney, muscle, and brain of control mice (Figure S1). Therefore, IS and PCS are likely to be transported into cells by OATs in other organs as well. However, further study is required to assess the relationship between uremic toxins and OATs in various tissues.

We found that AST-120 exerts different effects on IS and PCS levels. AST-120 was more effective in reducing PCS levels than IS levels. Because both IS and PCS originate in protein fermentation by colonic bacteria, their levels are modulated by intestinal factors such as prebiotics, probiotics, and laxatives [34,35,36,37]. The present findings are consistent with those of clinical studies reporting that prebiotics and probiotics were more effective at reducing PCS levels than IS levels [38,39]. A study in CKD rats showed that AST-120 administration decreased the production of uremic toxins and concurrently altered the composition of the gut microbiome [40]. Thus, the alterations in the intestinal environment caused by AST-120 reduce PCS levels more than IS levels. Moreover, a recent clinical study indicated that AST-120 may have additive effects on the continuous reduction of IS and PCS levels, but not of indoleacetic acid and hippuric acid, in patients undergoing hemodialysis [41]. It is very likely that the beneficial effect of AST-120 is mainly limited to uremic toxins originating from colonic microbiota metabolism.

In the present study, we used adenine-induced RF mice as our CKD model. The weight of several organs was much lower in RF mice than in control mice. In CKD patients, symptoms such as loss of appetite, sarcopenia, and metabolic acidosis have been observed. In our study, the body weight of RF mice fed 0.2% wt/wt adenine decreased over the 7 weeks of the study (Figure S2A). This decrease was in line with decreased food intake in this group (Figure S2B). We speculate that the decrease in food intake accounts for low-weight organs in RF mice. Additionally, the toxic effects of IS and PCS may also account for the decreased organ weight in RF mice.

Although previous studies have shown a renoprotective effect of AST-120 treatment in CKD rats [42,43], the effect has not been shown conclusively in clinical trials such as EPPIC-1 and EPPIC-2 that evaluated the effects of AST-120 on the progression of CKD when added to standard therapy [44,45,46]. Our study did not find a significant effect of AST-120 on renal function, which was comparable in the RF and RF + AST-120 groups; the expression of *Cyp1a1* did not change in the kidneys of RF mice following AST-120 treatment (Figure S3), although this may be attributable to our experimental model, in which severe renal damage had been induced before the start of AST-120 treatment, and to the effects of endogenous AhR ligands other than IS. However, we could evaluate the effect of AST-120 on the accumulation of uremic toxins because of the comparable renal function, since differences in renal function alter the degree of uremic toxin retention. The EPPIC-1 and EPPIC-2 trials only recorded the effect of AST-120 on renal function in CKD patients. However, as shown in the present study, accumulation of uremic toxins and its reduction by AST-120 can also affect systemic organs. Thus, the uremic toxin-reducing effect of AST-120 may contribute to protection of organs other than the kidney in CKD.

## 4. Conclusions

We found that the uremic toxins IS and PCS accumulate in whole-body organs in a mouse model of CKD. Attenuation of this accumulation by AST-120 is a potentially useful therapeutic strategy for systemic organ protection in CKD.

## 5. Materials and Methods

### 5.1. Animals

All animal experiments were approved by the Animal Committee of Tohoku University School of Medicine (2016PhA-019) on 8 February 2016. Experimental protocols and animal care were performed according to the guidelines for the care and use of animals established by Tohoku University. Male 8- to 9-week-old C57BL/6JJcl mice were purchased from CLEA Japan, Inc. (Tokyo, Japan). The mice were randomized to control and RF groups. Control-group mice were fed a normal diet (Oriental Yeast, Tokyo, Japan) for 7 weeks. RF-group mice were fed a diet containing 0.2% wt/wt adenine (Oriental Yeast) for 7 weeks to induce tubular injury [47], a model we used in previous work [17], with other mice. After 7 weeks, each group was further divided into two groups, one of which received 8% (wt/wt) AST-120 (Kremezin^®^; Kureha Pharmaceuticals, Tokyo, Japan). The concentration of AST-120 was selected based on findings from previous studies [25,48]. After 4 weeks, all mice were euthanized, and blood and organ samples were collected. Perfusion with saline was performed before organ collection. Blood samples were collected into EDTA-treated tubes.

### 5.2. Chemicals and Reagents

IS was purchased from Sigma-Aldrich (St. Louis, MO, USA). IS-d_4_ was purchased from Toronto Research Chemicals (Toronto, ON, Canada). PCS and PCS-d_4_ were synthesized by one of the authors (YM), and their identity and purity (>99%) were confirmed by nuclear magnetic resonance spectroscopy and elemental analyses (within ±0.3% of the theoretical values). Mouse monoclonal anti-IS antibodies (anti-IS antibody-producing hybridomas: 9A2F6) were obtained from Kureha Pharmaceuticals.

### 5.3. Histological Analysis and Immunohistochemistry

Kidney and skeletal muscle were fixed in 2% formaldehyde, embedded in paraffin, and sectioned. Tissue sections were subjected to hematoxylin and eosin staining and Elastica-Masson staining. Immunohistochemistry for IS was performed using a Histofine Simple Stain MAX PO (M) kit (Nichirei, Tokyo, Japan). Following deparaffinization, tissue sections were preheated in Cell Conditioning 1 (CC1) solution (Ventana Medical Systems, Inc., Tucson, AZ, USA) for 60 min at 90 °C, and then blocked by incubation with 0.3% hydrogen peroxide in methanol for 15 min. The section was incubated with Blocking Reagent A from a Histofine Simple Stain MAX PO (M) kit for 60 min at room temperature, followed by incubation with the anti-IS antibody (1:300) in phosphate-buffered saline containing 1% bovine serum albumin for 60 min at room temperature. Subsequently, the section was incubated with Blocking Reagent B from a Histofine Simple Stain MAX PO (M) kit for 10 min at room temperature. Bound antibodies were detected by treatment with the Simple Stain MAX PO (M) reagent for 20 min at 37 °C, using diaminobenzidine tetrahydrochloride as a substrate. Images were analyzed with ImageJ software (version1.46r, National Institutes of Health, Bethesda, MD, USA).

### 5.4. Sample Preparation for LC-MS/MS

Fifty-milligram tissue samples from kidney, heart, pancreas, WAT, BAT, testis, thymus, spleen, lung, small intestine, colon, and cecum were mixed with 400 µL of 0.1% formate methanol containing 2.5 µg/mL IS-d_4_ and 1 µg/mL PCS-d_4_, and homogenized for 30 s at 6000 rpm at room temperature using a Percellys 24 lysing and homogenization system (M&S Instruments Inc., Osaka, Japan). For muscle, brain, and liver, 800 µL of 0.1% formate methanol containing 2.5 µg/mL IS-d_4_ and 1 µg/mL PCS-d_4_ were added to 50 mg of tissue. After homogenization, samples were sonicated for 15 min and then centrifuged at 16,400× *g* for 20 min at 4 °C. An equal volume of 0.1% formate was added to the supernatant, and the mixture was analyzed by LC-MS/MS. For plasma, 150 µL of 0.1% formate methanol were added to 50 µL of plasma, and the mixture was vortexed for 1 s. After vortexing, samples were sonicated for 15 min, and then centrifuged at 16,400× *g* for 20 min at 4 °C. An equal volume of 0.1% formate was added to the supernatant, and the mixture was analyzed by LC-MS/MS.

### 5.5. LC-MS/MS Measurement of Uremic Toxins

Quantitative analysis of IS and PCS by LC-MS/MS was performed using a Nanospace SI-II HPLC platform (Shiseido, Tokyo, Japan) coupled to a TSQ Quantiva mass spectrometer (Thermo Fisher Scientific, Waltham, MA, USA), and operated in the negative mode. Each sample (3 µL) was injected onto a 100 × 2.0 mm Capcell Pak C18 MG III 3-µm column (Shiseido, Tokyo, Japan) with a flow rate of 0.3 mL/min. For gradient elution, mobile phase A was 10 mM ammonium acetate and mobile phase B was acetonitrile. Linear and stepwise gradients were programmed as follows: 0–1 min: 0–10% solvent B; 1–2 min: 10–40% solvent B; 2–3 min: 40–80% solvent B; 3–5 min: 80–100% solvent B; 5–7 min: 100% solvent B; 7–10 min: 0% solvent B. Quantification analyses by MS/MS were performed by a selected reaction monitoring mode, in which the transitions of the precursor ion to the product ion and collision energy (eV) were monitored: *m*/*z* 212→80, 21 eV for IS; *m*/*z* 216→80, 30 eV for IS-d_4_; *m*/*z* 187→107, 23 eV for PCS; *m*/*z* 191→111, 30 eV for PCS-d_4_. Spray voltage was 2500 V, vaporizer temperature was 320 °C, and ion transfer tube temperature was 350 °C. The intra- and inter-assay coefficients of precision for IS measurement were 8.3% and 2.5% at 0.78 μg/mL, respectively, and the intra- and inter-assay coefficients of accuracy were 1.8% and 5.1% at 0.78 μg/mL, respectively. Linearity was y = 0.00659804 + 0.000180261 × x, and the lower limit of detection was 0.39 μg/mL. The intra- and inter-assay coefficients of precision for PCS measurement were 10.6% and 3.7% at 0.78 μg/mL, respectively, and the intra- and inter-assay coefficients of accuracy were 10.7% and 6.0% at 0.78 μg/mL, respectively. Linearity was y = 0.00156406 + 6.83373e^−005^ × x, and the lower limit of detection was 0.2 μg/mL.

### 5.6. MSI of Kidney Sections

Kidney tissues were sectioned to 8-µm thickness with a cryostat and thaw-mounted onto indium tin oxide-coated glass slides. IS in the kidney was detected by the method we reported previously [17]. Six hundred milligrams of 9-aminoacridine were sublimated using iMLayer matrix sample preparation devices (Shimadzu, Kyoto, Japan), and the matrix thickness was monitored at 0.5 µm. After sublimation, recrystallization was carried out by methods described previously with slight modification [49]. A matrix-assisted laser desorption/ionization time-of-flight mass spectrometer (MALDI-TOF MS, AXIMA^®^ Confidence, Shimadzu) equipped with a 337-nm N_2_ laser was used for MALDI MSI analyses. Mass spectra were acquired in negative ionization mode and scanning mass range from *m*/*z* 50 to *m*/*z* 1000 in a high-resolution mode. Laser power, detection voltage, and accumulated number of MALDI-MSI images were 115, 3.0 kV, and 1/pixel, respectively. The spatial resolution in data points was 40 µm, giving 45,451 profiles over the entire region of the tissue section. Acquired MSI data were processed with the BioMap software package. The signal intensity of imaging data was represented as the normalized intensity.

### 5.7. Biochemical Measurements in Urine

Quantitative analysis of creatinine was performed using LC-MS/MS according to a method reported previously [50]. A Prominence LC-MS/MS system (Shimadzu, Kyoto, Japan) coupled to a TSQ Quantum Ultra mass spectrometer (Thermo Fisher Scientific, Waltham, MA, USA) was used. BUN was measured using a colorimetric detection kit (Arbor Assays, Ann Arbor, MI, USA). Urinary albumin concentration was determined using Albuwell M kits (Exocell Inc., Philadelphia, PA, USA) [51].

### 5.8. Quantitative PCR Analysis

A half-kidney segment was homogenized in ISOGEN (Nippon Gene Co., Ltd., Tokyo, Japan), and extracted according to the manufacturer’s directions. Extracted RNA was reverse-transcribed to cDNA using SuperScript III First-strand Synthesis SuperMix (Invitrogen, Carlsbad, CA, USA) according to the recommended protocol. Gene expression was measured with SYBR Premix Taq II (Takara, Kusatsu, Japan) with glyceraldehyde-3-phosphate dehydrogenase (*Gapdh*) as the reference gene, as previously described [17]. The list of primers is shown in Table S1.

### 5.9. Statistical Analysis

JMP Pro software version 12.2.0 (SAS Institute Inc., Cary, NC, USA) was used for statistical analysis. All values are expressed as box plots unless otherwise stated. Differences were considered statistically significant when *p* < 0.05. Statistical comparisons of multiple groups were made with ANOVA and the Tukey-Kramer test for normally distributed variables.

## Figures and Tables

**Table 1:** Mouse body and organ weights.

	Control (*n* = 6)	AST-120 (*n* = 6)	RF (*n* = 6)	RF + AST-120 (*n* = 6)	ANOVA *p* Value
Body weight (g)	25.5 ± 1.5	25.4 ± 1.6	20.7 ± 1.4 **^,††^	21.9 ± 1.2 **^,††^	<0.0001
Right kidney (mg)	147.2 ± 11.0	161.5 ± 12.8	74.6 ± 21.0 **^,††^	80.4 ± 11.6 **^,††^	<0.0001
Left kidney (mg)	136.5 ± 17.5	148.4 ± 13.5	67.3 ± 8.1 **^,††^	83.1 ± 17.9 **^,††^	<0.0001
Brain (mg)	457.9 ± 6.9	462.0 ± 11.5	431.9 ± 10.7 **^,††^	444.1 ± 14.5	0.0003
Heart (mg)	126.4 ± 13.2	126.9 ± 17.1	119.1 ± 8.0	135.3 ± 7.9	0.2
BAT (mg)	60.0 ± 23.1	73.1 ± 19.2	73.0 ± 19.6	69.7 ± 21.2	0.7
WAT (mg)	355.1 ± 137.5	374.8 ± 162.1	114.8 ± 18.3 **^,††^	112.9 ± 19.2 **^,††^	<0.0001
Liver (g)	1.05 ± 0.09	1.02 ± 0.07	0.93 ± 0.08	0.97 ± 0.09	0.08
Thymus (mg)	37.0 ± 7.5	36.8 ± 5.2	46.3 ± 7.6	45.5 ± 17.9	0.9
Lung (mg)	173.7 ± 53.5	168.2 ± 44.8	178.2 ± 51.2	186.3 ± 38.3	0.7
Pancreas (mg)	139.1 ± 18.8	133.1 ± 21.7	138.1 ± 19.7	135.4 ± 18.4	1
Spleen (mg)	54.3 ± 4.1	46.6 ± 9.3	59.5 ± 4.3 ^†^	66.3 ± 11.4 ^††^	0.002
Testis (mg)	215.6 ± 20.6	203.7 ± 37.8	159.6 ± 27.2 **^,†^	185.5 ± 10.8	0.005
Small intestine (mg)	807.2 ± 67.4	712.0 ± 71.4	749.2 ± 87.1	830.2 ± 100.2	0.08
Cecum (mg)	114.1 ± 18.0	101.8 ± 4.7	96.8 ± 23.3	112.2 ± 31.6	0.4
Colon (mg)	138.1 ± 20.3	138.3 ± 14.1	142.4 ± 38.0	150.8 ± 16.9	0.8

Data are expressed as means ± SEM. *n*, number of mice; BAT, brown adipose tissue; WAT, white adipose tissue; RF, renal failure. *p* values were derived using ANOVA for differences between groups. Tukey-Kramer test: ** *p* < 0.01 vs. control; ^†^ *p* < 0.05 vs. AST-120; ^††^ *p* < 0.01 vs. AST-120.

## Supplementary Materials

The following are available online at www.mdpi.com/2072-6651/10/1/19/s1, Figure S1: Relative mRNA expression levels of *Scl22a6* (organic anion transporter 1; OAT1) and *Slc22a8* (organic anion transpoter3; OAT3) in kidney, muscle, and brain of control mice (*n* = 6), Figure S2: Changes in body weight and food intake, Figure S3: Relative mRNA expression levels of *Cyp1a1* in kidney, Table S1: Primers used in PCR analysis.
