# Supplementary material for: Impact of the Oral Adsorbent AST-120 on Organ-Specific Accumulation of Uremic Toxins: LC-MS/MS and MS Imaging Techniques
Source: Toxins (Basel). 2017 Dec 28;10(1):19. doi: 10.3390/toxins10010019 (PMC5793106; doi:10.3390/toxins10010019)
Supplement: Supplementary file 1 [file toxins-10-00019-s001.pdf]

Supplementary Materials: Impact of the Oral Adsorbent AST-120 on Organ-Specific Accumulation of Uremic Toxins: LC-MS/MS and MS Imaging Techniques

Emiko Sato, Daisuke Saigusa, Eikan Mishima, Taeko Uchida, Daisuke Miura, Tomomi Morikawa-Ichinose, Kiyomi Kisu, Akiyo Sekimoto, Ritsumi Saito, Yuji Oe, Yotaro Matsumoto, Yoshihisa Tomioka, Takefumi Mori, Nobuyuki Takahashi, Hiroshi Sato, Takaaki Abe, Toshimitsu Niwa and Sadayoshi Ito

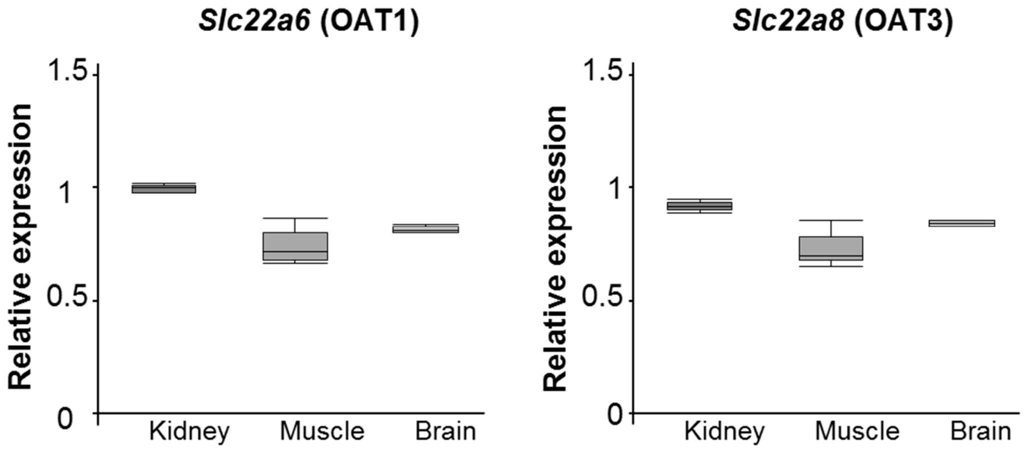

Figure S1. Relative mRNA expression levels of *Scl22a6* (organic anion transporter 1; OAT1) and *Slc22a8* (organic anion transpoter3; OAT3) in kidney, muscle, and brain of control mice (n = 6). Data are shown as box plots. The data are normalized to *Gapdh* expression levels.

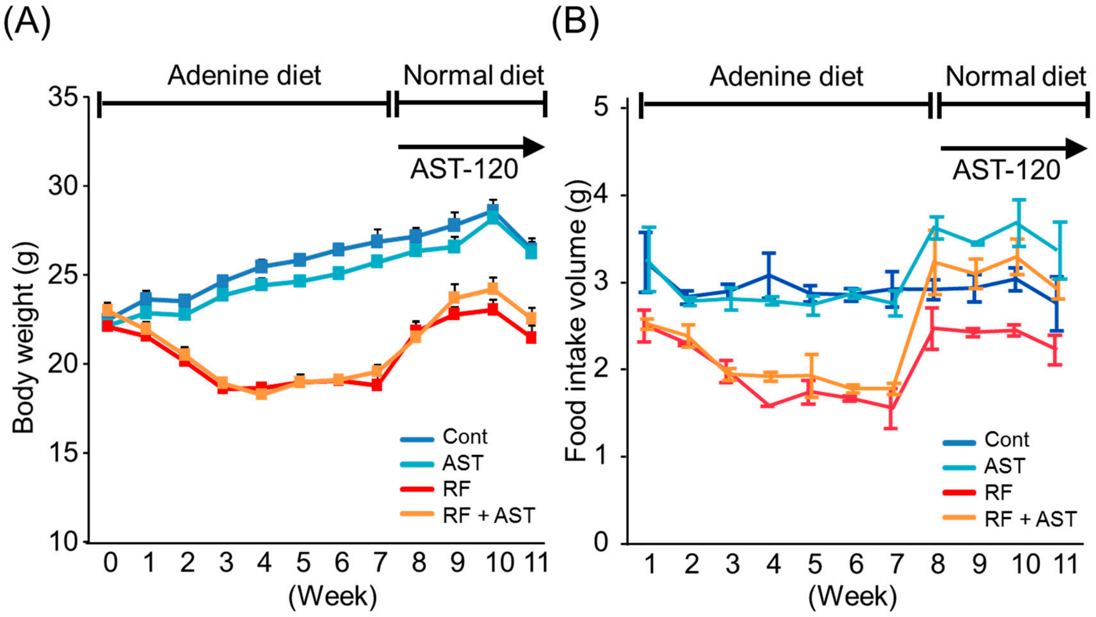

Figure S2. Changes in body weight and food intake. (**A**) Body weight changes, and (**B**) food intake volume changes among 11 weeks. Control group mice were fed a normal diet for 7 weeks. Renal-failure mice were fed a diet containing 0.2% wt/wt adenine for 7 weeks to induce tubular injury. After 7 weeks, each group was further divided into two groups, one of which received 8% (wt/wt) AST-120. After 4 weeks, all mice were euthanized. Cont, control (n = 6); AST, AST-120 (n = 6); RF, renal failure (n = 7); RF + AST, RF mice treated with AST-120 (n = 6).

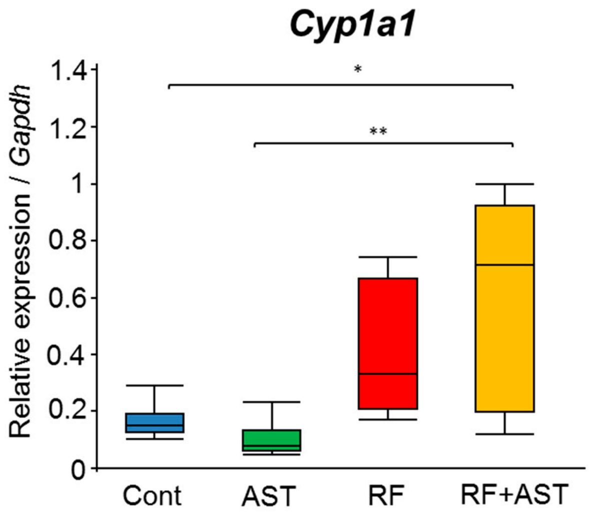

Figure S3. Relative mRNA expression levels of *Cyp1a1* in kidney. Data are shown as box plots. Tukey-Kramer test: * $p < 0.05$, ** $p < 0.01$. Cont, control ($n = 6$); AST, AST-120 ($n = 6$); RF, renal failure ($n = 7$); RF + AST, RF mice treated with AST-120 ($n = 5$).

Table S1. Primers used in PCR analysis.

**Gene**	**Sequence (5′ → 3′)**	
Pai-1	Fw	TTCAGTGGCCAATGGAAGACTCCT
	Rv	AGGGCAGTTCCACAACGTCATACT
Cyp1a1	Fw	GGCCACTTTGACCCTTACAA
	Rv	CAGGTAACGGAGGACAGGAA
Slc22a6	Fw	CAGTCAGTGTGTCAGGGACCTTGTA
	Rv	TGTGTGGAACCTGGAATGATGAG
Slc22a8	Fw	GCCAGGACACTCAGCTTGGA
	Rv	GCAGTCATTAGCTCTGTGGTTGATA
